# Functional profile of S100A4‐deficient T cells

**DOI:** 10.1002/iid3.85

**Published:** 2015-09-25

**Authors:** Kathleen Weatherly, Marie Bettonville, David Torres, Arnaud Kohler, Stanislas Goriely, Michel Y. Braun

**Affiliations:** ^1^ Institute for Medical ImmunologyUniversité Libre de Bruxelles (ULB)GosseliesBelgium

**Keywords:** Ca‐binding protein, T cell, motility, inflammation

## Abstract

The protein S100A4 is best known for its significant role in promoting motility and invasive capacity of cancer cells. Since S100A4 expression has been reported also in T cells, we analyzed its potential role in T cell motility and inflammation. Using *S100a4^+/Gfp^* mice, we show here that S100A4 is exclusively expressed by memory T cells of CD4^+^ or CD8^+^ subpopulations, predominantly of the effector memory T cell subtype. However, the protein was not required for in vitro memory T cell migration toward gradients of the inflammatory chemokine CXCL10. Moreover, T cell memory response was normal in S100A4‐deficient mice and lack of *S100a4* gene expression did not induce any defect in promoting the development of protective immunity or inflammatory reactions leading to autoimmunity. Taken together, our results demonstrate that S100A4 activity is dispensable for T cell motility/migration and inflammatory potential.

## Introduction

S100A4 belongs to the vast family of S100 proteins. One of the characteristics shared by all S100 proteins is the presence of a pair of calcium‐binding helix‐loop‐helix domains (EF‐hand calcium‐binding regions), present at either end of the proteins 1. Binding of calcium to these motifs results in a conformational change that exposes a hydrophobic patch through which S100 proteins bind and regulate the activity of other molecules, since they themselves do not exhibit enzymatic activity. S100A4 has been shown to interact with proteins involved in the cytoskeletal architecture, such as tropomyosin, and to F‐actin, which could have a possible link with motility 2, 3. More recently, intracellular S100A4 was also shown to interact with the Rho binding and regulating protein, Rhotekin 4. This protein has been intimately linked with cell polarity and migration. However, the cytoskeletal molecules with which intracellular S100A4 interacts and have received the most attention is undoubtedly the non‐muscle myosin heavy chains. These molecules are essential in cellular processes where movement is required. Recent studies indicate that S100A4 preferentially binds to and inhibits the assembly of non‐muscle myosin‐α filaments 5, 6, 7. Consistent with these observations, absence of intracellular S100A4 leads to an over‐assembly of non‐muscle myosin‐α complexes and the inhibition of chemotactic motility in cultured bone marrow‐derived macrophages 8. It should also be noted that motility‐promoting effects of S100A4 might not be exclusively due to direct regulation of the cytoskeleton. The Wnt/β‐catenin pathway is also associated with S100A4‐mediated cell migration 9, where the presence of a T cell factor (TCF) binding site in the 5′‐untranslated region of the S100A4 promoter has been identified. Furthermore, β‐catenin has been shown to bind to this region and consequently increases the expression of the intracellular S100A4 protein, resulting in subsequent enhanced migration and invasion of cancer cells 10. Finally, S100A4 could also play a role in cell motility as a soluble extracellular mediator since the addition of recombinant molecules in cultures has been reported to promote cellular migration in endothelial cells, smooth muscle cells, T lymphocytes and fibroblasts 11, 12, 13, 14. S100A4 is supposed to promote its chemo‐attracting activity through interactions with specific receptors, such as the receptor for advanced glycation endproducts (RAGE) 15, 16.

Many studies have reported S100A4 expression in T cells 17, 18, 19. Since motility is an intrinsic part of T cell function, we hypothesized that S100A4 could represent an important modulator of T cell migration in immune responses. We report here that S100A4 is specifically expressed by memory T cells. However, the protein is not required for in vitro T cell migration toward chemokine gradients, and *S100a4^Gfp/Gfp^* knockin mice showed normal capacity to generate memory T cell response and immunity, demonstrating that S100A4 activity is dispensable for T cell motility.

## Methods

### Mice

All mice used in this study were on a C57BL/6 background. *S100a4^+/Gfp^* mice (B6.129S6‐S100a4^tm1Egn^) 20 were purchased from the Jackson Laboratory (Bar Harbor, USA). In these mice, parts of exon 2 and 3 of the endogenous gene were replaced by in‐frame sequence encoding Green Fluorescent Protein. *S100a4^Gfp/Gfp^* mice were obtained by crossing heterozygous mice. Genotyping was carried out by PCR on purified tail DNA samples using specific primers for wild‐type *S100a4* and *Gfp* genes 20. *Rag2^−/−^* mice were obtained from the Jackson Laboratory and C57BL/6 mice were purchased from Charles River. All mice used in this study were issued from breeding pairs housed in specific pathogen‐free conditions (FELASA) at the Institute for Medical Immunology (Gosselies, Belgium). Experimental animal protocols were performed in accordance with the Animal Care and Use Committee guidelines of the Université Libre de Bruxelles.

### T cell purification

CD4^+^ T cells were purified from spleens by magnetic‐activated cell sorting (Dynal CD4^+^ T cell negative isolation kit, Invitrogen, Gent, Belgium) according to the manufacturer's protocol. Naive or memory T cells were purified from previously isolated CD4^+^ T cells subsets by positive or negative selection of CD62L‐expressing cells using the magnetic cell sorting kit (CD62L microbeads, Miltenyi Biotec, Bergisch Gladbach, Germany) according to the manufacturer's protocol. For Treg differentiation experiments, CD25‐positive cells were removed from purified CD4^+^ T cells by magnetic‐activated cell sorting using FITC‐conjugated anti‐CD25 antibodies and anti‐FITC microbeads (Miltenyi Biotec).

### Cell culture

For anti‐CD3/CD28‐mediated stimulation of purified T cells, 96‐flat‐bottomed‐well plates were coated for 2 h at 37°C with 5 μg/ml of anti‐CD3 (BD Biosciences, Erembodegem, Belgium) in PBS. Purified naive T cells were plated at a concentration of 1.5 × 10^6^ cells per ml and stimulated in the presence of 2 μg/ml anti‐CD28 (BD Biosciences) for 3 days.

### T cell differentiation

For differentiation of naive CD4^+^ T cells into different Thelper subsets, 96‐flat‐bottomed‐well plates were coated for 2 h at 37°C with 5 μg/ml of anti‐CD3 (145‐2C11, BD Biosciences) in PBS. Purified naive CD4 T cells from C57/BL6 WT or *S100a4^Gfp/Gfp^* mice were plated at a concentration of 1.5 × 10^6^ cells per ml and stimulated in the presence of 2 μg/ml anti‐CD28 (37.51, BD Biosciences) and different combinations of cytokines (all from R&D Systems, Abingdon, UK) and antibodies (all from BD Biosciences) in RPMI 1640, 2 mM l‐Glutamine, 25 mM Hepes medium and supplemented with 1 mM sodium pyruvate, 0.1 mM nonessential amino acids, 100 U/ml penicillin, 100 μg/ml streptomycin (all from Lonza, Petit Rechain, Belgium) and 10% FCS (PAA Laboratories, Pasching, Austria). For Th0 differentiation, cells were cultured with 10 μg/ml anti‐IFN‐γ and 10 μg/ml anti‐IL‐4. For Th1 differentiation, cells were stimulated in presence of 10 ng/ml IL‐12 and 10 μg/ml anti‐IL‐4. For Th2 differentiation, cells were stimulated with 10 ng/ml IL‐4 and 10 μg/ml anti‐IFN‐γ. For Th7 differentiation, cells were stimulated in presence of 10 ng/ml IL‐6, 10 ng/ml IL‐23, 5 ng/ml TGFβ, 10 μg/ml anti‐IFNγ, 10 μg/ml anti‐IL2, and 10 μg/ml anti‐IL‐4. For Treg differentiation, cells were stimulated with TGFβ (5 ng/ml) and 20 U/ml IL‐2. After 3 days of culture, intracellular staining for IL‐17 and IFN‐γ were performed (see protocol below). Commercially available enzyme‐linked immunosorbent assay (ELISA) kits were used according to the manufacturer's protocol (Duoset ELISA, R&D systems) for the detection of murine IL13 in culture supernatants.

### Western blot analysis

SDS polyacrylamide gel (SDS–PAGE) and immunoblotting were performed according to standard procedures. Briefly, cells were lysed by RIPA lysis buffer (Santa Cruz, Heidelberg, Germany) on ice. Cell lysates with equal amounts of proteins (15 μg) were separated in 12% SDS–PAGE. Separated proteins were then electrophoretically transferred to a polyvinylidene difluoride membrane (GE Healthcare, Diegem, Belgium), which was subsequently blocked at 4°C for 1 h with 5% non‐fat dry milk in TBST (20 mM Tris, pH 7.6, 137 mM NaCl, 0.1% Tween 20). The blots were then incubated with appropriate dilutions of primary antibodies overnight at 4°C in TBST containing 5% nonfat dry milk (for GAPDH) or 5% BSA (for S100A4). Primary antibodies used for Western blot analysis include rat polyclonal antibody for S100A4 (dilution 1:1000, Abcam, Cambridge, UK), and mouse monoclonal antibody for GAPDH (dilution 1:2000, Meridian Life Science, Memphis, USA). After three washes with TBST, the blots were incubated with horseradish peroxidase‐conjugated secondary antibodies either against rat (dilution 1:1000, GE Healthcare) or against mouse (dilution 1:2000, BD Biosciences) in TBST with 5% milk. After several washes with TBST, the blots were incubated at room temperature for 5 min with ECL (Lumigen, Southfield, USA). This was followed by detection with the ChemiDoc XRS (BioRad Laboratories, Temse, Belgium) and quantified by software analysis (Quantity One 4.5.1, Biorad Laboratories). Antibodies against GAPDH were used as loading controls.

### Transwell migration assay

Purified memory CD4^+^ T cells from control and *S100a4^Gfp/Gfp^* mice were labeled with 1 µM carboxy‐fluorescein diacetate succinimidyl‐ester (CFSE, Molecular Probes/Invitrogen) according to the manufacturer's instructions. CFSE labeling was assessed by flow cytometry. Labeled T cells (7 × 10^5^) were added to the top chamber of the transwell (6.5 mm Corning® Transwell® with 5.0 µm pore polycarbonate membrane insert, Sigma–Aldrich, Diegem, Belgique). The lower chamber contained medium with 50 or 100 ng/ml CXCL10 (CXC chemokine 10, eBiosciences) as a chemo‐attractant. Cells were allowed to migrate for 2 h and 30 min at 37°C in the presence or absence of chemokine. Migrated CFSE positive T cells were then collected from the bottom well and were quantified by flow cytometry on a CyAn ADP LX 9 Color (Beckman Coulter, Suarlée, Belgium). A fixed number of unlabeled WT CD4^+^ T cells (2 × 10^5^) was added in the bottom compartment of each well as a standard for determining the relative number of CFSE‐labeled cells that migrated through the membrane, as previously described 21.

### Experimental autoimmune encephalomyelitis (EAE)

One day before EAE induction, female *Rag2^−/−^* mice were intravenously injected with 5 × 10^6^ purified CD4^+^ T cells from control or *S100a4^Gfp/Gfp^* female mice. Reconstituted female *Rag2^−/−^* mice were subcutaneously injected with 100 μg of MOG35–55 (myelin oligodendrocyte glycoprotein, Sigma) in IFA (Incomplete Freund's Adjuvant, Difco, Erembodegem, Belgium) containing 400 μg of Mycobacterium tuberculosis (Difco). On the same day and again 48 h later, mice received an intraperitoneal injection of 500 ng of pertussis toxin (Sigma). Mice were scored, blinded, as follows: 0: healthy, no symptoms; 1: limp tail; 1.5: flaccid tail and impaired righting reflex; 2: limp tail and inability to right itself when placed on its back; 2.5: one hind leg paralyzed; 3: partial hind limb paralysis with one or both hind limbs dragging but some movement preserved; 3.5: both hind legs completely paralyzed; 4: complete hind limb paralysis with or without moderate forelimb weakness; 5: complete hind limb paralysis with forelimb involvement—moribund—breathing appears labored. Animals were monitored and weighted daily and given fluids (0.5 ml NaCl 0,9%, intraperitoneally) if signs of dehydration were observed and as a routine if they reached a score of 4.

For the isolation of CNS‐infiltrating leukocytes, brains were removed and incubated in RPMI 1640 media containing collagenase D (2.5 mg/ml, Roche, Vilvoorde, Belgium) and DNase I (100 µg/ml, Roche) for 30 min at 37°C with rotation. Homogenates were then passed through a 70 µm cell strainer. Brain leukocytes were then isolated by centrifugation on 30/70 Percoll (GE Healthcare Life Sciences) gradients.

### L. Monocytogenes infection

For effector responses, adult *S100a4^+/+^* controls, *S100a4^+/Gfp^* or *S100a4^Gfp/Gfp^* mice were immunized intraperitoneally with 5 × 10^5^ CFU of L. Monocytogenes (Lm) actA‐OVA. After 60 days, mice were challenged intravenously with 5 × 10^5^ CFU/mouse of Lm‐OVA. Five days after challenge, number of OVA‐specific CD8^+^ T cells was determined. In brief, peripheral blood cells were labeled with an OVA‐specific pentamer (3 μl/50 μl of blood, R‐PE Labelled Pro5 MHC Pentamer, F093‐2C‐E, ProImmune) 15 min at room temperature (dark) then cells were labeled with 50 μL of a mix of anti‐CD8, ‐CD62L, ‐CD44, ‐CD4, and ‐CD19 for 20 min at 4°C. Red blood cells were lysed by FACS_TM_ lysing solution buffer (BD Biosciences) during 10 min. Cells were then washed and fixed by 1.5% paraformaldehyde (VWR).

IFN‐γ production by CD8^+^ T cells was measured by intracellular staining. Briefly, spleen cells were harvested and cultured at 37°C with OVA_257‐264_ peptide and IL‐2 (10 ng/ml) in complete culture medium. For intracellular staining, GolgiPlug (1 μl/1 × 10^6^ cells, BD Biosciences) was added after 2 h. Cells were then harvested 2 h later and stained with anti‐CD3, ‐CD8, ‐CD44, and ‐CD62L antibodies. They were fixed and permeabilized with Cytofix/Cytoperm solution (BD Biosciences) and labeled with anti‐IFN‐γ antibody before analysis.

### Colitis

CD4^+^ T cell subsets were purified from spleens of control and *S100a4^Gfp/Gfp^* female mice. CD4^+^ T cells were then stained with anti‐CD4, ‐CD25, and ‐CD45RB fluorescent monoclonal antibodies. CD4^+^ CD25^−^ CD45RB^high^ fractions were then sorted on a MoFlo (Dakocytomation) with a purity of greater than 98%. To induce colitis, *Rag2^−/−^* female mice were injected intraperitoneally with 100 μl of NaCl 0.9% containing 5 × 10^5^ control or *S100a4^Gfp/Gfp^* CD4^+^ CD25^−^ CD45RB^high^ T cells. Changes in body weight were assessed twice weekly.

### Flow cytometry

For cell surface staining, cells were resuspended in PBS with 1% heat‐inactivated FCS and were incubated for 30 min in the dark at 4°C with fluorescent monoclonal antibodies. For intracellular cytokine staining, cells were resuspended in RPMI medium containing 50 ng/ml of PMA (Phorbol 12‐myristate 13‐acetate, Sigma) and 500 ng/ml of Ionomycin (Sigma) for 4 h. GolgiPlug (1 μl/1 × 10^6^ cells, BD Biosciences) were added after 2 h incubation. Cell were stained for surface protein and then for IFN‐γ and IL‐17 using the BD Cytofix/Cytoperm™ Kit (BD Biosciences) according to the manufacturer's protocol. For FOXP3 staining, the cells were stained following the manufacturer's instructions of the FOXP3 Staining Buffer Set kit (eBiosciences). The following anti‐mouse mAbs were purchased from BD Biosciences: PB‐conjugated anti‐CD4, PE‐conjugated anti‐CD4, PerCP‐conjugated anti‐CD4, PB‐conjugated anti‐CD8, PerCP‐conjugated anti‐CD8, PerCP‐conjugated anti‐CD3, APC‐conjugated anti‐B220, APC‐conjugated anti‐CD44, PeCy5‐conjugated anti‐CD44, PE‐conjugated anti‐CD62L, PB‐conjugated anti‐CD62L, PeCy7‐conjugated anti‐CD19/CD4, PB‐conjugated anti‐FOXP3, APC‐conjugated anti‐IFNγ, PE‐conjugated anti‐IFNγ, and PE‐conjugated anti‐IL17. Immunostained cells were fixed by 1,5% paraformaldehyde (VWR) and analyzed on a FACS Cyan ADP LX 9 color (Beckam Coulter) using Summit version 4.3 software (DakoCytomation).

### Statistical analysis

The statistical analyses were performed using Graphpad Prism (Graphpad Software, Inc.). Mann–Whitney *U* test was applied. Differences were considered significant at *P *< 0.05.

## Results

### T cell development in S100A4‐deficient mice

**Figure 1:**
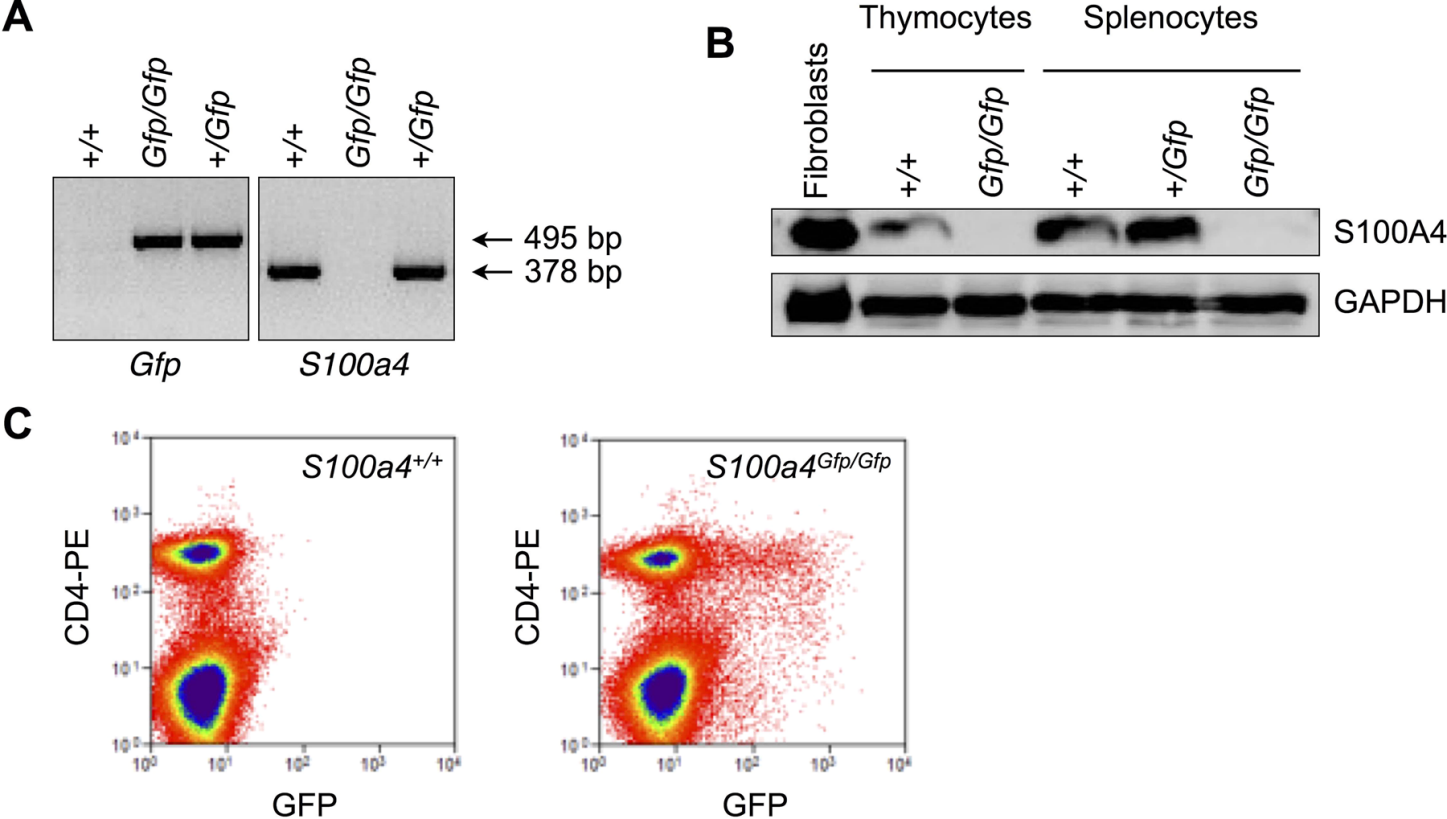
S100A4 expression in B6.*S100a4^Gfp/Gfp^* mice. A: Genes coding for GFP or wild‐type S100A4 were detected by PCR on DNA isolated from B6 mice, heterozygous B6.S100a4^*+/Gfp*^ or homozygous B6.S100a4^*Gfp/Gfp*^. B: Western blot analysis of S100A4 protein expression on spleen cell or thymocyte lysates isolated from mice as in A. Positive control for S100A4 expression included lysate from NIH‐3T3 fibroblasts. C: GFP expression in spleen CD4^+^ T cells isolated from *S100a4^Gfp/Gfp^* or *S100a4^+/+^* mice was analyzed by flow cytometry.

**Figure 2:**
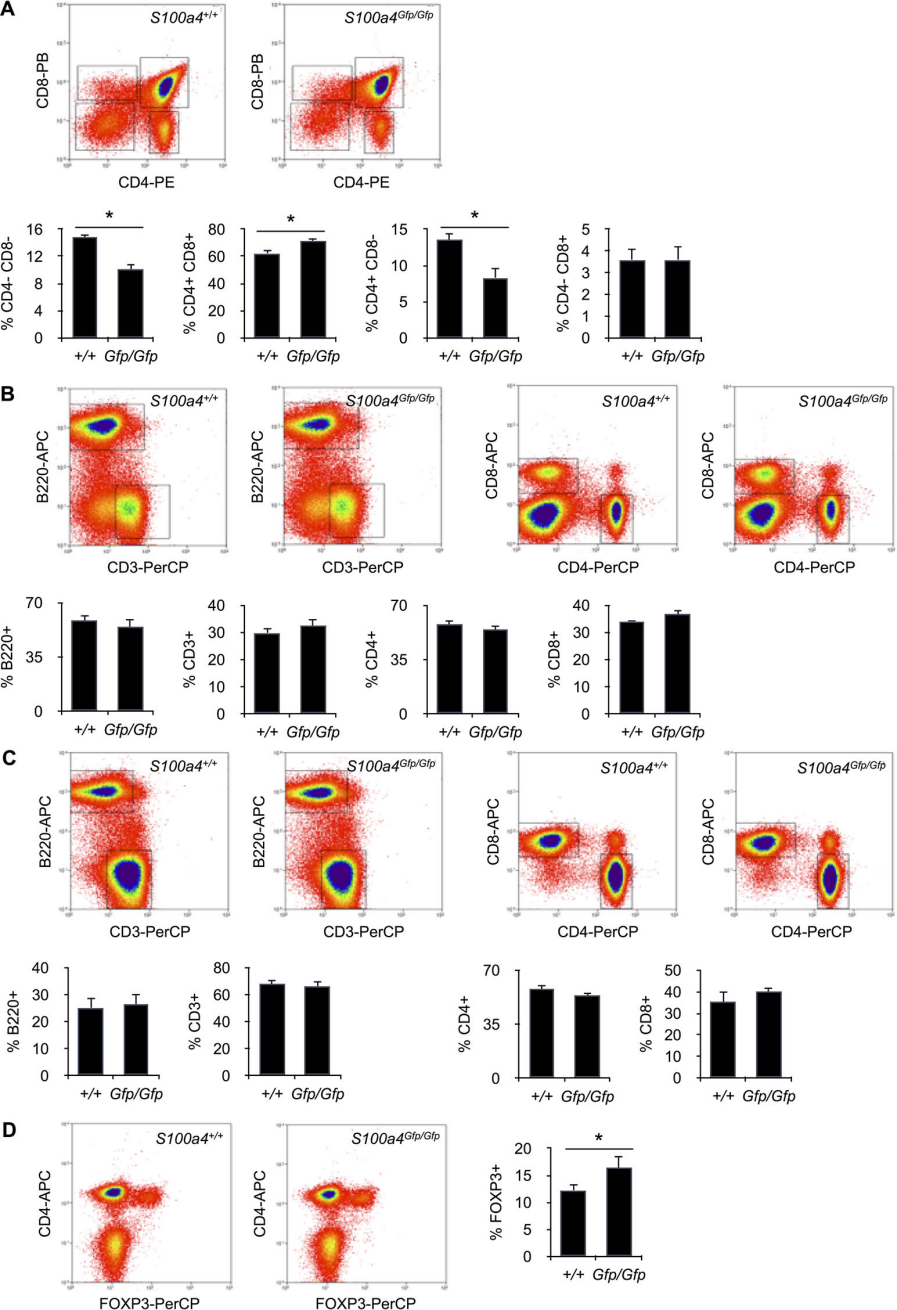
T cell subsets in mice lacking S100A4 expression. A: Subsets of thymocytes in S100a4^Gfp/Gfp^ or S100a4^+/+^ mice were identified by flow cytometry. Relative numbers of CD4^−^ CD8^−^ DN, CD4^+^ CD8^+^ DP, CD4^+^ CD8^−^ SP, and CD4^−^ CD8^+^ SP cells in thymocytes isolated from S100a4^Gfp/Gfp^ or S100a4^+/+^ mice. Mean ± SD. *n* = 5 mice per group. *P < 0.05. B: Subsets of spleen cells in S100a4^−/−^ or S100a4^+/+^ mice were identified by flow cytometry. Relative numbers of B220^+^ B cells as well as CD3^+^, CD4^+^, or CD8^+^ T cells in spleen cells isolated from S100a4^Gfp/Gfp^ or S100a4^+/+^ mice. Mean ± SD. *n* = 5 mice per group. C: Subsets of lymph node cells in S100a4^−/−^ or S100a4^+/+^ mice were identified by flow cytometry. Relative numbers of B220^+^ B cells as well as CD3^+^, CD4^+^, or CD8^+^ T cells in lymph node cells isolated from S100a4^Gfp/Gfp^ or S100a4^+/+^ mice. Mean ± SD. *n* = 5 mice per group. D: Natural FOXP3^+^ T regulatory cells were identified by flow cytometry. Relative numbers of FOXP3^+^ T cells within splenic CD4^+^ CD3^+^ T cells isolated from S100a4^Gfp/Gfp^ or S100a4^+/+^ mice. Mean ± S *n* = 5 mice per group. *P < 0.05.

Using *S100a4^Gfp/Gfp^* knockin mice, we have investigated the role played by S100A4 in T cell differentiation and function. In these animals, part of *S100a4* gene has been replaced by *Gfp* (Fig. 1A). Consequently, S100A4 protein expression was not detected by Western Blotting in thymocytes and spleen cells from *S100a4^Gfp/Gfp^* mice (Fig. 1B). On the other hand, GFP expression was seen in T cells purified from the spleen of *S100a4^Gfp/Gfp^* animals, confirming the replacement of endogenous S100A4 protein with GFP (Fig. 1C). In the thymus, GFP was only expressed by CD11b^+^ CD4^−^ CD8^−^ double negative thymocytes, suggesting that S100a4 expression was limited to non‐T cell populations (data not shown). Nevertheless, experiments were conducted to see whether deficiency in S100A4 expression could alter thymic selection. As depicted in Figure 2A, *S100a4^Gfp/Gfp^* mice exhibited a limited reduction in the relative number of immature CD4^−^ CD8^−^ double negative thymocytes as well as in mature CD4^+^ single positive T cells. Accordingly, these lower frequencies were compensated by an increase in the relative number of CD4^+^ CD8^+^ double positive thymocytes. The relative numbers of single CD8^+^ thymocytes did not differ between wild‐type mice and *S100a4^Gfp/Gfp^* mice. Despite differences observed in thymocyte subpopulations, the overall process of T cell maturation and differentiation was not modified by the absence of a functional S100A4 and normal numbers of T cells were found in peripheral secondary lymphoid organs of *S100a4^Gfp/Gfp^* animals (Fig. 2B and C). However, we noticed that S100a4^*Gfp/Gfp*^ mice contained relatively more FOXP3^+^ CD25^+^ CD4^+^ T regulatory cells (Fig. 2D), but this did not lead to noticeable signs of immune dysfunction and S100a4^*Gfp/Gfp*^ mice developed normally and appeared healthy, with body weight comparable to that exhibited by S100A4‐sufficient mice.

### S100A4 expression is restricted to memory T cells

**Figure 3:**
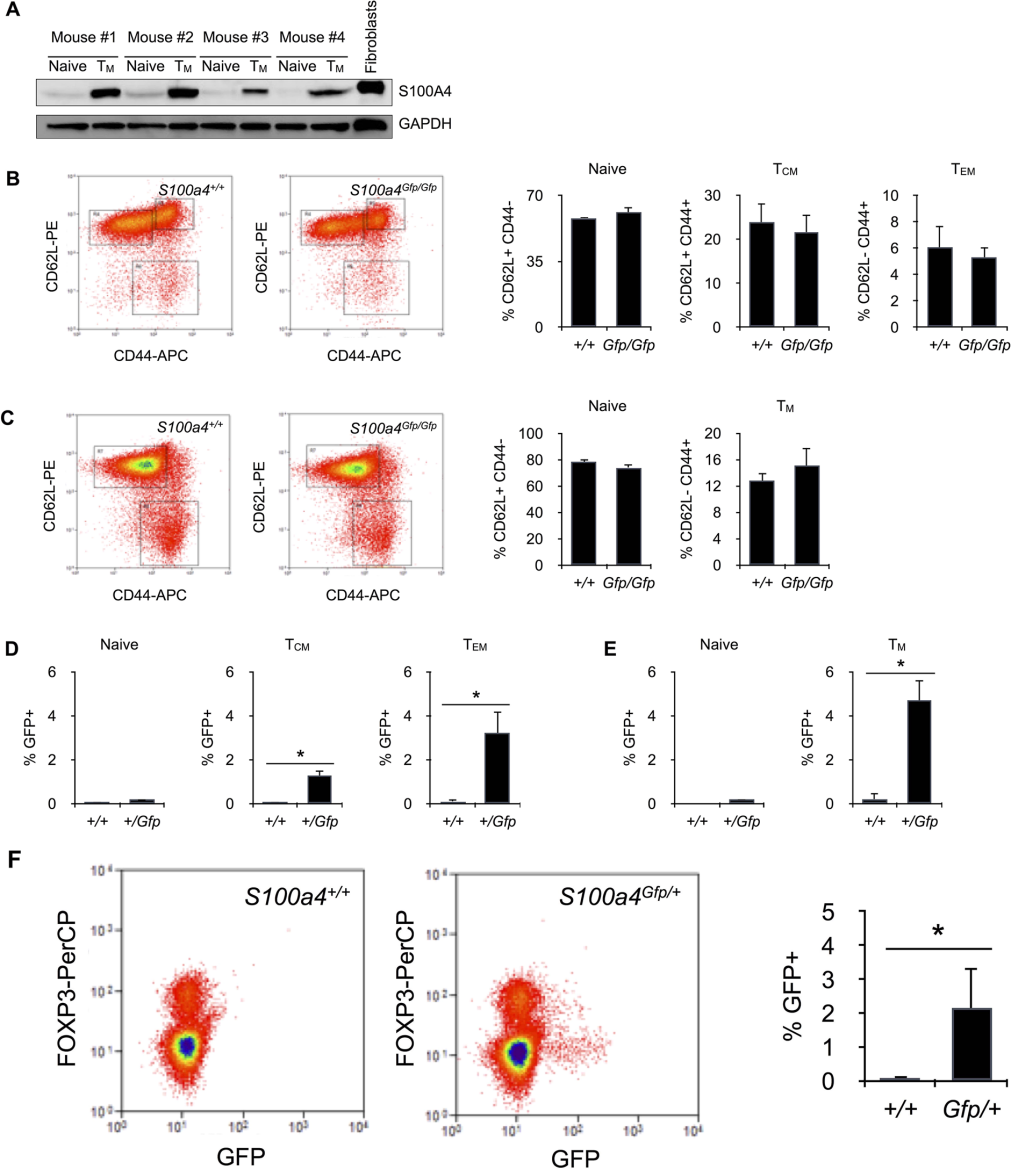
Memory T cells express S100A4. A: Western blot analysis of S100A4 expression in purified naive or memory CD4^+^ T cells. Positive control for S100A4 expression included lysate from NIH‐3T3 fibroblasts. B: Subsets of central (T_CM_) and effector (T_EM_) memory CD8^+^ T cells were identified in the spleen of *S100a4^Gfp/Gfp^* or *S100a4^+/+^* mice by flow cytometry. Relative numbers of CD44^low^ CD62L^high^ naive T cells, CD44^high^ CD62L^high^ T_CM_ and CD44^high^ CD62L^low^ T_EM_ within CD3^+^ CD8^+^ T cells isolated from *S100a4^Gfp/Gfp^* or *S100a4^+/+^* mice. Mean ± SD. *n* = 5 mice per group. C: Subsets of memory CD4^+^ T cells were identified in the spleen of *S100a4^Gfp/Gfp^* or *S100a4^+/+^* mice. Relative numbers of CD44^low^ CD62L^high^ naive T cells and CD44^high^ CD62L^low^ memory T cells within CD3^+^ CD4^+^ T cells isolated from *S100a4^Gfp/Gfp^* or *S100a4^+/+^* mice. Mean ± SD. *n* = 5 mice per group. D: S100A4 expression in naive, central (T_CM_) and effector (T_EM_) memory CD8^+^ T cells. Relative numbers of S100A4‐expressing cells (% GFP^+^ cells) within CD44^low^ CD62L^high^ naive, CD44^high^ CD62L^high^ T_CM_, or CD44^high^ CD62L^low^ T_EM_ CD3^+^ CD8^+^ T cells were determine by flow cytometry. Mean ± S *n* = 5 mice per group. *P < 0.05. E: S100A4 expression in naive and memory CD4^+^ T cells. Relative numbers of S100A4‐expressing cells (% GFP^+^ cells) within CD44^low^ CD62L^high^ naive or CD44^high^ CD62L^low^ T_EM_ CD3^+^ CD4^+^ T cells were determine by flow cytometry. Mean ± SD. *n* = 5 mice per group. *P < 0.05.

Since S100A4 has been reported to be expressed in antigen‐experienced T cells 17, 18, 19, we looked at S100A4 expression in memory T cells. As shown in Figure 3A, S100A4 was detected by Western Blotting mostly in isolated memory T cells. Memory CD8^+^ T cells can be divided in two subsets depending on their expression of CD44 and CD62L molecules. Central memory CD8^+^ T cells (T_CM_), which are mostly located in secondary lymphoid organs, are defined as CD44^high^ CD62L^high^ cells. Whereas effector memory CD8^+^ T cells (T_EM_) are CD44^high^ CD62L^low^ and, therefore, cannot home to lymph nodes. In CD4^+^ T cells, expression of CD44 and CD62L does not allow to identify clearly T_CM_ from T_EM_. We will only refer to CD44^high^ CD62L^low^ memory CD4^+^ T cells, as opposed to CD44^low^ CD62L^high^ naive CD4^+^ T cells. There was no difference in the relative numbers of CD8^+^ T_CM_ and CD8^+^ T_EM_ cells between S100a4^*Gfp/Gfp*^ mice and *S100a4^+/+^* mice (Fig. 3B). This was also observed for memory CD4^+^ T cells (Fig. 3C). Levels of GFP expression in cells isolated from *S100a4^+/Gfp^* mice, which, in these animals, is directly controlled by *S100a4* promoter, give information on the expression of S100A4 protein itself. As depicted in Figure 3D and E, GFP^+^ cells were only present in the memory T cell compartments, and this was observed for both CD4^+^ and CD8^+^ T cells. However, there was more GFP^+^ cells in T_EM_ CD8^+^ T cells (about 3.5%) then in T_CM_ CD8^+^ T cells (about 1.2%; Fig. 3D). Thus, we could conclude from these observations that S100A4 was exclusively expressed by memory T cells, and that, under steady‐state conditions, S100A4 expression correlated with the development of effector function within memory T cells. Since the lack of S100A4 increased moderately the number of FOXP3^+^ CD4^+^ T cells (Fig. 2D), we also analysed GFP expression in Treg cells from *S100a4^+/Gfp^* mice. As depicted in Figure 3F, only 2–3% of FOXP3^+^ CD4^+^ T cells were GFP‐positive and, presumably, expressed S100A4.

### Normal differentiation and function in S100A4‐deficient T cells

**Figure 4:**
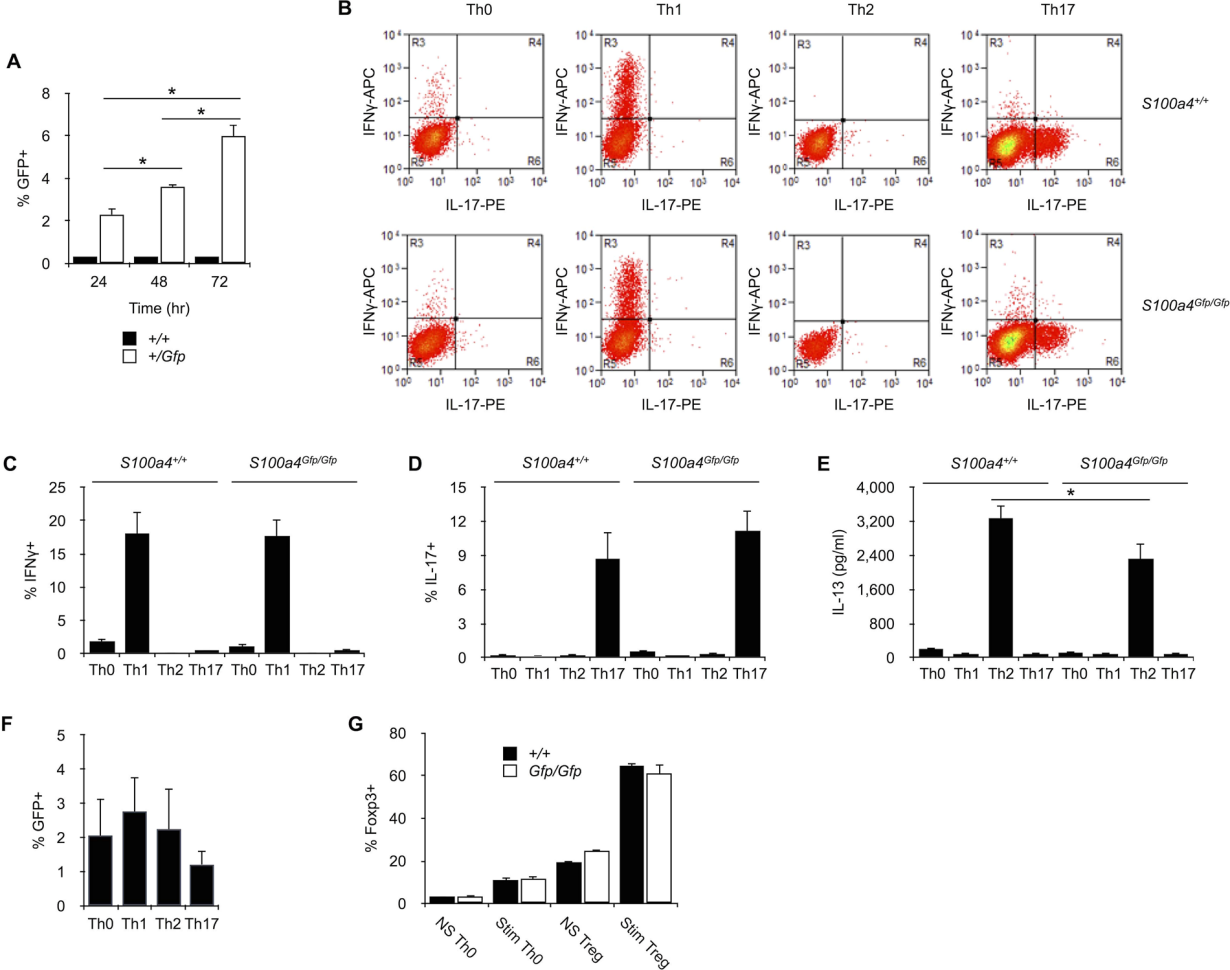
Thelper differentiation in S100A4‐deficient mice. A: S100A4 expression after T cell activation. Splenic CD4^+^ T cells were purified from *S100a4^+/Gfp^* or *S100a4^+/+^* and stimulated with immobilized anti‐CD3 and soluble anti‐CD28 antibodies. After 24, 48, or 72 h of culture, relative numbers of S100A4‐expressing cells (% GFP^+^ cells) were determine by flow cytometry. Mean ± SD. *n* = 5 culture replicates. *P< 0.05. Data are representative of two independent experiments. B: Th0, Th1, Th2, or Th17 differentiation in *S100a4^Gfp/Gfp^* or *S100a4^+/+^* CD4^+^ T cells. Splenic CD4^+^ T cells were purified from *S100a4^Gfp/Gfp^* or *S100a4^+/+^* mice and stimulated with immobilized anti‐CD3 and soluble anti‐CD28 antibodies in Th0‐, Th1‐, Th2‐, or Th17‐conditioning medium. IFNγ^+^ or IL‐17^+^ cells within CD3^+^ CD4^+^ cells were identified by flow cytometry. C: Relative numbers of IFNγ^+^ cells within *S100a4^+/Gfp^* or *S100a4^+/+^* Th0, Th1, Th2, or Th17 cells. Mean ± SD. *n* = 5 culture replicates. *P < 0.05. Data are representative of two independent experiments. D: Relative numbers of IL‐17^+^ cells within *S100a4^+/Gfp^* or *S100a4^+/+^* Th0, Th1, Th2, or Th17 cells. Mean ± S *n* = 5 culture replicates. Data are representative of two independent experiments. E: Amount of IL‐13 present in the supernatants of Th0, Th1, Th2, or Th17 cell cultures was assessed by ELISA. Mean ± SD. *n* = 5 culture replicates. *P < 0.05. Data are representative of two independent experiments. F: S100A4 expression in Th0‐, Th1‐, Th2‐, or Th17‐derived CD4^+^ effector T cells was analyzed by flow cytometry. Mean ± SD. *n* = 5 culture replicates. Data are representative of two independent experiments. G: FOXP3^+^ Treg cell differentiation in S100a4^Gfp/Gfp^ or S100a4^+/+^ CD25^−^ CD62L^+^ CD4^+^ naive T cells. Splenic T cells were purified from S100a4^Gfp/Gfp^ or S100a4^+/+^ mice and stimulated (Stim) or not (NS) with immobilized anti‐CD3 and soluble anti‐CD28 antibodies in Th0‐ or Treg‐conditioning media. Relative numbers of FOXP3+ T cells within CD3+ CD4+ cells were identified by flow cytometry. Mean ± SD. *n* = 6 culture replicates. Data are representative of two independent experiments.

Since S100A4 expression was not seen in naive T cells, we investigated whether T cell activation could increase its expression. Purified *S100a4^+/Gfp^* CD4^+^ T cells were stimulated in vitro with a combination of immobilized anti‐CD3 and soluble anti‐CD28 antibodies and, after 1, 2, and 3 days, GFP^+^ T cells were counted by flow cytometry. As seen in Figure 4A, CD3‐mediated activation modestly increased the number of GFP^+^ cells. Thus, S100A4 expression can be induced in a small proportion of T cells after activation.

It has been reported that soluble S100A4 shifts the Th1/Th2 balance of differentiating T cells toward Th2 phenotype by inhibiting of Th1 cell differentiation 22. Another study has shown that the lack of S100A4 expression caused a deficit in Th17 cell differentiation and the development of arthritis 23. We next investigated whether the lack of S100A4 expression could modify the capacity of CD4^+^ T cells to differentiate into subtypes of effector cells. *S100a4^Gfp/Gfp^* or *S100a4^+/+^* CD4^+^ T cells were purified and cultured in conditions that drive differentiation of CD4^+^ T cells toward Th0, IFN‐γ‐secreting Th1, IL‐13‐secreting Th2 or IL‐17‐secreting Th17 cells. As seen in Figure 4B–E, *S100a4^Gfp/Gfp^* CD4^+^ T cells were able to differentiate into Th0, Th1, or Th17 cells to the same extent seen in *S100a4^+/+^* CD4^+^ T cells. Though we were able to differentiate *S100a4^Gfp/Gfp^* Th2 cells, we noticed a slight reduction of IL‐13 in culture supernatants of Th2‐differentiated *S100a4^Gfp/Gfp^* T cells (Fig. 4E). S100A4 expression status as assessed by GFP expression in CD4^+^ T cells was similar regardless of culture condition (Fig. 4F). Taken together, our results clearly indicate that S100A4 expression in T cells does not appear to be required for the differentiation of T helper subsets. Since cytometry analysis of T cell subsets in the spleen of *S100a4^Gfp/Gfp^* mice revealed a significant increase in the relative number of FOXP3^+^ CD4^+^ T cells (Fig. 2D), we analyzed the potential role played by S100A4 in the differentiation of Treg cells. As depicted in Figure 4G, the lack of S100A4 expression did not modify the capacity of naive CD4^+^ T cells to differentiate into FOXP3^+^ T cells when cultured in conditions that drive differentiation of CD4^+^ T cells toward Treg cells.

**Figure 5:**
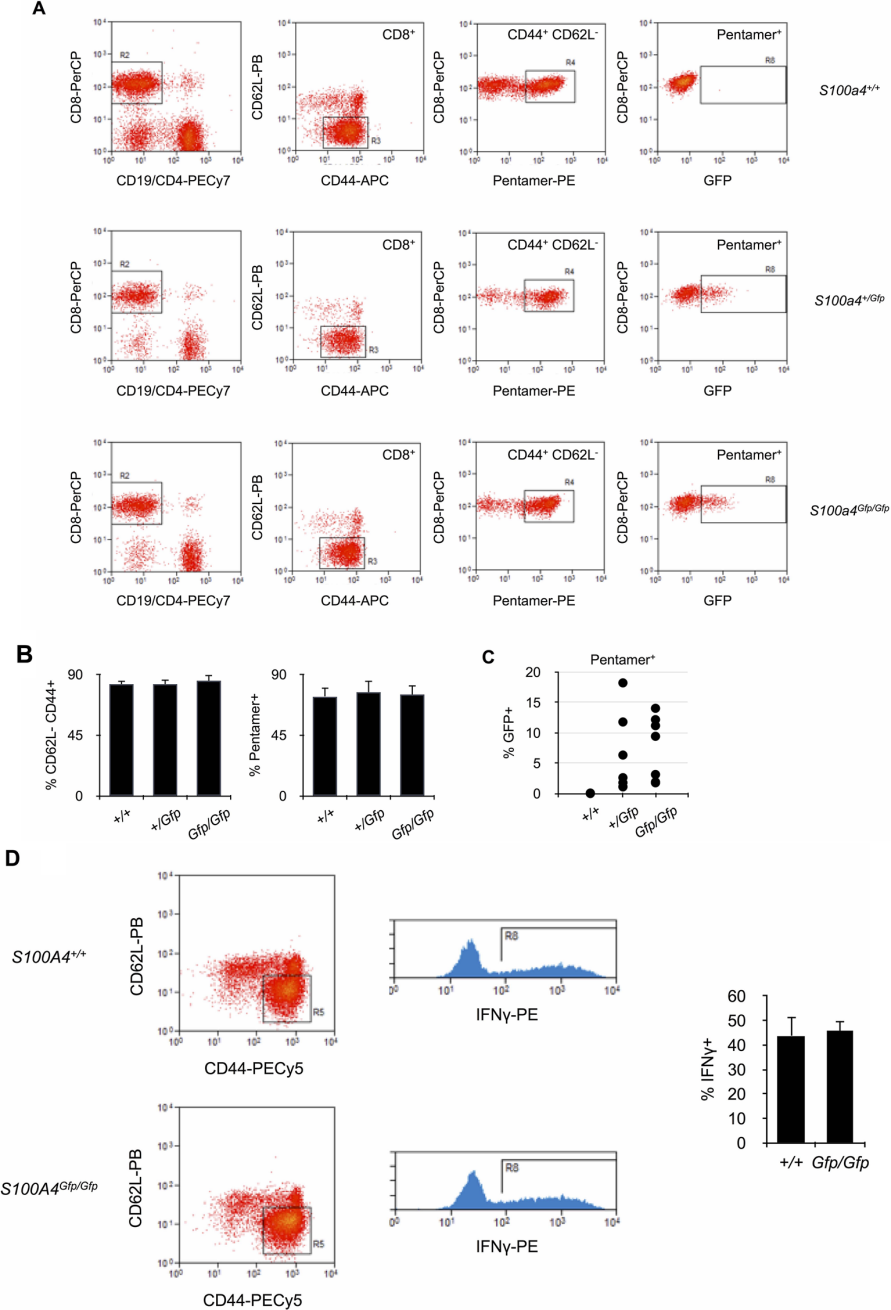
Effector T cell differentiation in S100A‐deficient mice. A: Analysis of OVA‐specific T cell response in *S100a4^Gfp/Gfp^*, *S100s4^+/Gfp^*, or *S100a4^+/+^* mice after infection with OVA‐trangenic *L. monocytogenes*. OVA‐H‐2K^b^ pentamer‐positive CD44^+^ CD62L^−^ GFP^+^ CD8^+^ T cells were identified by flow cytometry. B: Relative numbers of OVA‐H‐2K^b^ pentamer‐positive CD44^+^ CD62L^−^ effector CD8^+^ T cells isolated from *S100a4^Gfp/Gfp^*, *S100s4^+/Gfp^*, or *S100a4^+/+^* mice after infection with OVA‐trangenic *L. monocytogenes*. Mean ± SD. *n* = 10 mice per group. C: Relative numbers of S100A4^+^ cells (% GFP^+^) within pentamer^+^ CD44^+^ CD62L^−^ CD8^+^ effector T cells isolated from *S100a4^Gfp/Gfp^*, *S100s4^+/Gfp^*, or *S100a4^+/+^* mice after infection with OVA‐trangenic *L. monocytogenes*. Mean ± SD. *n* = 10 mice per group. D: IFNγ production by CD44^+^ CD62L^−^ CD8^+^ effector T cells isolated from *S100a4^Gfp/Gfp^* or *S100a4^+/+^* mice after infection with OVA‐trangenic *L. monocytogenes*. T cells were isolated and restimulated in vitro with OVA for 16 h before flow cytometry analysis. Relative number of IFNγ is given (right panel). Mean ± S *n* = 10 mice per group.

We also analyzed the effect of S100A4 expression on effector T cell differentiation in vivo. *S100a4^Gfp/Gfp^*, *S100a4^+/+^*, or *S100a4^+/Gfp^* animals were infected with attenuated ActA‐deficient OVA‐transgenic *L. monocytogenes*. Eight weeks later, mice were challenged with OVA and OVA‐specific CD8^+^ T cells were observed by flow cytometry after labeling the cells with PE‐coupled OVA‐H‐2K^b^ pentamers. As depicted in Figures 5A and B, the relative numbers of OVA‐specific CD44^high^ CD62L^low^ CD8^+^ effector T cells were comparable 5 days after OVA challenge in all immunized animals. Thus, lack of S100A4 did not appear to modify the capacity of CD8^+^ T cells to differentiate into effector T cells. S100A4 expression, as assessed by measuring the percentage of GFP^+^ CD8^+^ T cells, was observed in OVA‐specific effector T cells (Fig. 5C). Whether the lack of S100A4 expression could modify the function in effector T cells was investigated and *S100a4^Gfp/Gfp^* CD8^+^ T cells from infected mice were assessed for their capacity to synthesized IFN‐γ after antigenic stimulation. As seen in Figure 5D, *S100a4^+/+^* cells and *S100a4^Gfp/Gfp^* cells were equally able to produce IFN‐γ, demonstrating that S100A4 expression was dispensable for the function of effector CD8^+^ T cells.

### S100A4 deficiency does not alter the motility and the capacity to mediate autoimmune inflammation in T cells

**Figure 6:**
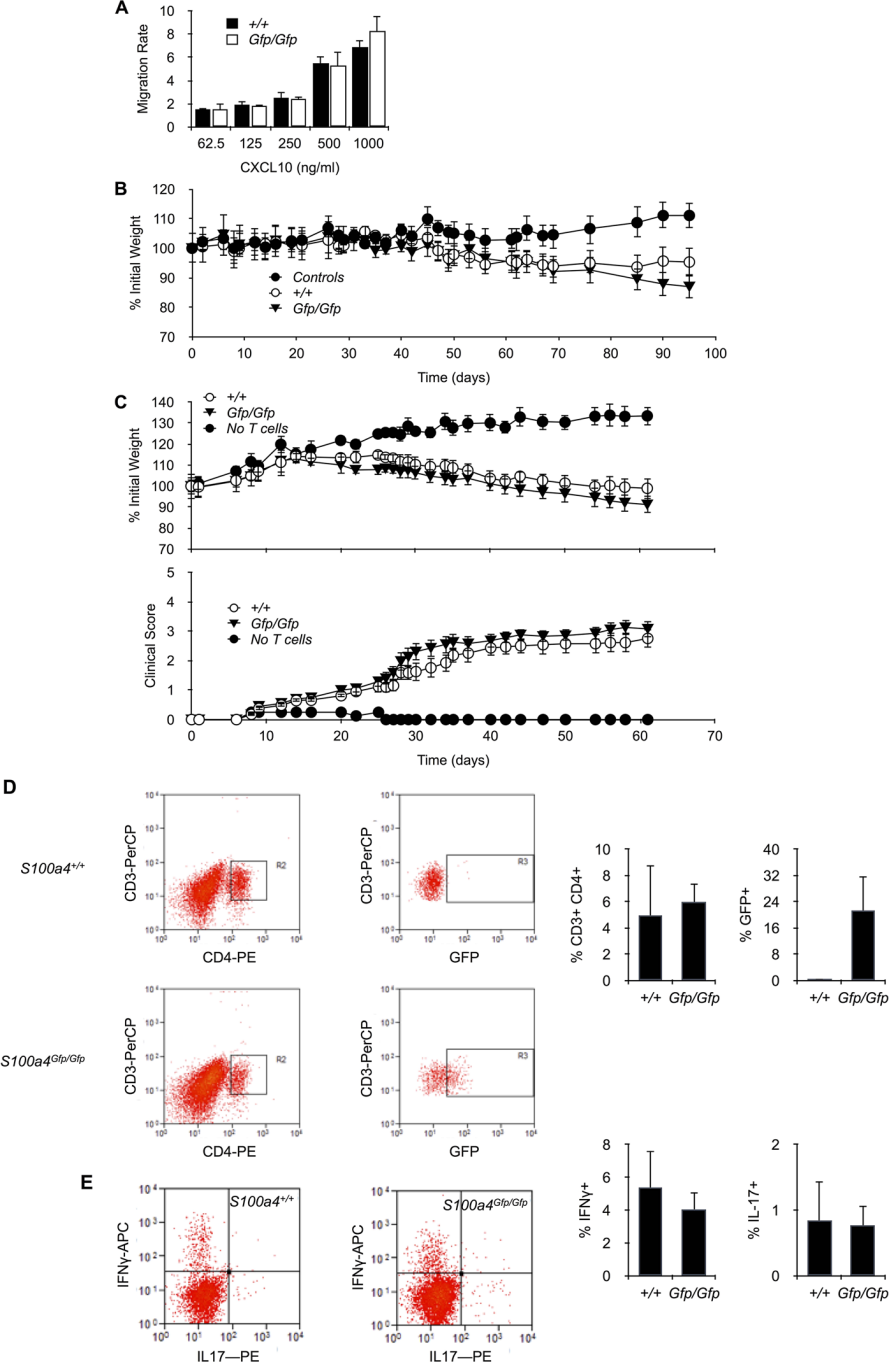
S100A4 expression in CD4^+^ T cells is not required for the motility of T cells and the development of autoimmune inflammation. A: The capacity of purified *S100s4^Gfp/Gfp^* or *S100a4^+/+^* memory CD4^+^ T cells to migrate toward gradients of CXCL10 concentration was carried out in a transwell migration assay (see Methods). Mean ± SD. *n* = 5 culture replicates. Data are representative of four independent experiments. B: Percent of initial weight in *Rag2^−/−^* mice adoptively transferred with CD45RB^high^ CD4^+^ T cells isolated from *S100s4^Gfp/Gfp^* or *S100a4^+/+^* mice. *n* = 10 mice per experimental group. C: Percent of initial weight (upper panel) or EAE clinical score (lower panel) in *Rag2^−/−^* mice adoptively transferred with MOG‐specific CD4^+^ T cells isolated from MOG‐immunized *S100s4^Gfp/Gfp^* or *S100a4^+/+^* mice. *n* = 10–12 mice per experimental group. D: *Rag2^−/−^* mice adoptively transferred with MOG‐specific CD4^+^ T cells isolated from MOG‐immunized *S100s4^Gfp/Gfp^* or *S100a4^+/+^* mice, were harvested and CD4^+^ CD3^+^ T cells in the brains were analyzed by flow cytometry to determine their relative number in cellular isolates, as well as to detect among them the frequency of GFP‐expressing cells. Mean ± S *n* = 5 mice for the group transferred with *S100a4^+/+^* CD4^+^ T cells and *n* = 6 mice for the group transferred with *S100a4^Gfp/Gfp^*. E. *Rag2^−/−^* mice adoptively transferred as in D. CD4^+^ T cells in the brain were analyzed by flow cytometry to detect cells expressing IL‐17A and IFN‐γ. Mean ± SD. *n* = 5 mice for the group transferred with *S100a4^+/+^* CD4^+^ T cells and *n* = 6 mice for the group transferred with *S100a4^Gfp/Gfp^*.

S100A4 has been described to modulate the motility of macrophages 8. This property is thought to be the direct consequence of the capacity of S100A4 to regulate the assembly of non‐muscle myosin‐α filaments which is required for cell motility 21, 24. We, therefore, analyzed the capacity of S100A4 protein to modulate the motility of memory T cells. Cell motility is required for a functional immune system and T cell migration to sites of infection is an important step of effective immune responses. This process is usually regulated by gradients of soluble chemokines which inform T cells on the location of infection sites. We, therefore, analyzed in vitro the capacity of CD62L^−^ CD44^+^ memory CD4^+^ T cells lacking the expression of S100A4 to migrate toward gradients of the chemokine CXCL10. As seen in Figure 6A, the lack of S100A4 expression did not modify the capacity of T cells to migrate toward CXCL10. Thus, S100A4 in T cells does not appear to be required for cell motility. However, one should not forget that, according to our results, only a small fraction of memory T cells (3–5%) express S100A4 (Fig. 3D and E). This could explain why the lack of S100A4 expression does not impact T cell migration in our assay.

We also analyzed the role played by S100A4 in regulating immune responses where T cell migration represented a critical step. Two models of autoimmune diseases were investigated. First, *S100a4^Gfp/Gfp^* CD45RB^high^ CD4^+^ T cells were analysed for their capacity to mediate autoimmune colitis after transfer into lymphopenic congenic *Rag2^−/−^* recipients as described previously 25. As depicted in Figure 6B, animals adoptively transferred with *S100a4^Gfp/Gfp^* or *S100a4^+/+^* CD45RB^high^ CD4^+^ T cells developed equally colitis as assessed over time by the loss of body weight. Moreover, *S100a4^Gfp/Gfp^* CD4^+^ T cells were also analyzed for their capacity to mediate the development of experimental autoimmune encephalomyelitis (EAE) in *Rag2^−/−^* recipients immunized with MOG protein 26. Again, we did not observe any modification in the capacity of T cells to mediate autoimmunity that could have been caused by the lack of S100A4 expression and mice adoptively transferred with *S100a4^Gfp/Gfp^* CD4^+^ T cells developed EAE to the same extent as those receiving *S100a4^+/+^* CD4^+^ T cells (Fig. 6C). Moreover, relative numbers of *S100a4^Gfp/Gfp^* or *S100a4^+/+^* CD4^+^ T cells present in the CNS‐infiltrating cell population were similar (Fig. 6D). Since the production of IFNγ and IL‐17 by CNS‐infiltrating CD4^+^ T cells is critical for the development of EAE 27, we also analysed the production of these cytokines in cells isolated from the brain of mice that developed EAE. As seen in Figure 6E, the relative numbers of IFNγ‐ or IL‐17‐producing cells among *S100a4^Gfp/Gfp^* and *S100a4^+/+^* T cells were not significantly different. Thus, the loss of S100A4 expression did not modify the capacity of T cells to migrate to target organs and mediate locally autoimmunity. Interestingly, however, GFP expression was noticed in about 20% of *S100a4^Gfp/Gfp^* T cells infiltrating the brain of mice that developed EAE (Fig. 6D). Therefore, since S100A4 has been recently detected in CNS CD4^+^ T cells in EAE 28, it can be concluded that S100A4 is expressed in CNS‐infiltrating T cells but is redundant for T cell trafficking to the CNS.

## Discussion

S100A4 is known to modulate the motility of both non‐transformed and cancer cells by regulating the localization and stability of cellular protrusions. This is believed to occur through its ability to bind to the C‐terminal end of the non‐muscle myosin‐IIA heavy chain coiled‐coil and to disassemble myosin‐IIA filaments 5, 6, 7. Since T cell motility depends heavily on myosin II A‐regulated adhesions 21, 24, we investigated the hypothesis that S100A4 was critical for T cell motility and trafficking through tissues. We first observed that only memory T cells did express S100A4 and, among them, the sub‐population of effector memory T cells (T_EM_) had the highest proportion of S100A4‐positive cells. However, our observations that, like wild‐type T cells, S100A4‐deficient memory T cells were able to migrate in vitro toward chemokine gradients and could mediate autoimmune inflammatory disorders, demonstrated that S100A4 was not required for their motility. This conclusion is in sharp contrast to what has been observed for the motility of macrophages, where the loss of S100A4 expression promoted over‐assembly of myosin IIA filaments, defects in chemokine‐stimulated motility and reduced recruitment to sites of inflammation 8. Much of the difficulty in elucidating the precise function of S100 protein family has been attributed to a possible functional redundancy and compensation by its conserved family members 1. The S100 protein family is indeed the largest subgroup within the superfamily of proteins carrying the Ca2^+^‐binding EF‐hand motif which consists of at least 25 members. Thus, one possible explanation for the lack of phenotype in S100A4‐deficient T cells could be the functional replacement of S100A4 by one or several S100 family member(s). In this context, S100P may constitute a good candidate to replace S100A4 in T cells. Both S100A4 and S100P have indeed been reported to bind to non‐muscle myosin IIA directly and to be capable of unzipping myosin IIA/actin filaments to permit changes in cellular filopodial projections in order to provide the necessary motive force 29. Moreover, like S100A4, S100P has been reported to bind RAGE and activate cancer cell proliferation and invasion 30, 31. However, S100P expression has yet to be documented in T cells.

Ambartsumian and co‐workers have shown that S100A4 protein mediates the attraction of T cells in vitro and that S100A4 production by cancer cells stimulates tumor infiltration by T cells 13. These observations support the hypothesis that S100A4 can act as a soluble factor capable of stimulating the migration of T cells. Since most of our experiments were conducted with purified T cells, one could hypothesize that the lack of phenotype accompanying S100A4 deficiency in T cells could be due to poor S100A4 production (indeed, in our hands, less than 6% of memory T cells expressed S100A4). On the contrary, cancer cells, fibroblasts and macrophages express strongly S100A4 1, 8, 14 and could constitute good extracellular sources of it for stimulating in vivo T cell motility and migration. Our results would indicate, however, that S100A4, produced by other cells than T cells, is not necessary for the development of T cells nor for the generation of memory T cells since S100A4‐deficient mice exhibit a normal distribution of peripheral T cell subsets and were able to differentiate antigen‐specific memory T cells to the same extent as that observed in wild‐type mice.

It has also been reported that S100A4 protein alters the expression of transcription factor and signal transduction pathway genes involved in the T‐cell lineage differentiation. T cells challenged with soluble S100A4 demonstrated reduced proportion of Th1‐polarized cells shifting the Th1/Th2 balance toward the Th2 pro‐tumorigenic phenotype 22. However, S100A4 capacity to alter the Th1/Th2 balance of differentiating T cells was shown to be the result of S100A4‐mediated inhibition of Th1 polarization rather than a direct effect on Th2 cell differentiation. Functional studies of human Th2 cells as well as mouse models of allergy showed that deletion of S100A4 resulted in decreased signs of allergy including Th2 cell activation, humoral immunity, and infiltration of effector cells 32. Taken together, these observations would support the concept that S100A4 plays a critical role in the differentiation of Th cell subsets and would be more specifically required for Th2 cell differentiation. In our own studies, we noticed indeed that S100A4‐deficient T cells placed in Th2‐conditioned cultures produced slightly more IL‐13, a specific Th2 cytokine, than S100A4‐sufficient T cells. We would like to emphasize, however, that, in our hands, S100A4 deficiency did not prevent Th1 cell differentiation nor the development of Th1/Th17‐mediated autoimmune inflammation and diseases.

S100A4 expression has also been reported to have an effect on Th17 helper cell differentiation. Brisslert and co‐workers have noted that S100A4‐deficient mice are less sensitive to induced autoimmune arthritis and exhibit a deficit in T cell capacity to produce IL‐17 after anti‐CD3 stimulation 23. Since our results clearly indicate that S100A4‐deficient CD4^+^ T cells can be differentiated in vitro into Th17, one must suppose that the deficit in IL‐17 production observed by Brisslert and co‐workers in S100A4‐deficient T cells could not be attributed to an intrinsic defect of Th17 differentiation. Interestingly, these authors also observed a slight increase in the relative number of FOXP3^+^ CD4^+^ T cells in S100A4 mice. Whether increased Treg‐mediated immunoregulation could explain the deficit in IL‐17 production and lower susceptibility to autoimmune disease observed in S100A4‐deficient animals remains to be explored.

In conclusion, since we did not observe fundamental immune dysfunction in mice lacking S100A4 expression, our data strongly support the notion that S100A4 shares its properties with other S100 proteins in immune cells and, more specifically, in T cells. This is an important finding since S100A4 has been shown to be an important mediator of cancer cell metastasis and therapeutic targeting of its function is a major strategy being currently developed against cancer 33. In light of our results, specific inhibition of S100A4 could be safely applied in complementing anti‐tumor vaccination.

## Conflict of Interest

The authors declare no competing financial interests.
